# Patella Fracture After Total Knee Arthroplasty: A Review

**DOI:** 10.7759/cureus.53281

**Published:** 2024-01-31

**Authors:** Konstantinos Tsivelekas, Dimitrios Pallis, Stavros Lykos, Evangelos Triantafyllou, Petros Nikolakakos, Anastasia Tilentzoglou, Stamatios A Papadakis

**Affiliations:** 1 Second Department of Orthopaedics, KAT General Hospital of Attica, Athens, GRC

**Keywords:** tka, patella periprosthetic fracture, patella fracture, periprosthetic fracture, total knee arthroplasty

## Abstract

The management and underlying causes of patellar periprosthetic fractures (PPF) after total knee arthroplasty (TKA) constitute an issue of growing importance given the rising frequency of these procedures. Patella periprosthetic fractures, though relatively rare, pose significant challenges and are a frequent indication for revision surgeries. Despite a decrease in overall incidence, PPFs remain the second most common type of periprosthetic fractures after TKA. Several factors have been identified and associated with patient-specific factors, surgical technique errors, and implant-related causes. Currently extensor apparatus integrity, bone stock, and component loosening are the major concerns and indications for the selective treatment approach. In this study, a thorough review of the existing literature was performed summarizing the epidemiology, clinical manifestation, treatment approach, and functional outcome of PPF. This review aims to underline the significance of such predisposing factors, point out the severity of PPF, and offer insights into the optimal intra- and post-operative management of the patella.

## Introduction and background

Total knee arthroplasty (TKA) is one of the most common joint replacement surgeries [1]. More than a million TKAs are being performed annually, which is mainly attributed to the rise of osteoporosis, obesity, and aging of the population [2,3]. As the frequency of TKAs continues to escalate, it becomes increasingly crucial to prevent complications such as periprosthetic fractures around the knee joint [4].

**Figure 1:**
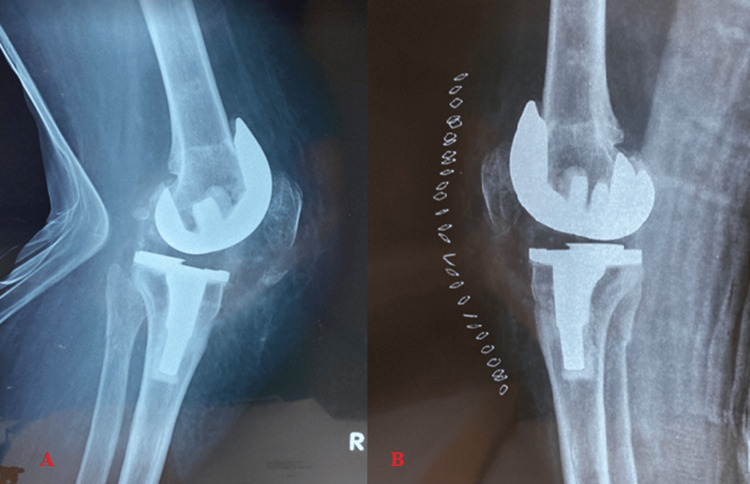
Non-traumatic patella periprosthetic fracture in a 65-year-old female patient of our clinic, identified during the 2-year routine follow-up. The patient claimed severe anterior knee pain and loss of extension. A) Detection of the fracture of the distal pole of the patella in lateral knee X-ray. B) Immediate postoperative lateral knee X-ray after internal fixation of the fracture using a 1.5 mm bone tunnel with bone sutures. Written informed consent was signed by the patient to publish the X-rays.

For over 30 years, the complications arising from total knee arthroplastys (TKAs) have been an orthopedic challenge [5]. Brick and Scott in 1988 identified patellofemoral complications including periprosthetic fractures, dislocation, implant damage, osteonecrosis, aseptic loosening, and patellar impingement, as the main responsible reason for almost 50% of revision TKA surgeries [6]. However, recent decades have witnessed a reduction in these complications. Patellar issues now represent approximately 2-20% of all TKA complications. Notably, they have been identified as major causes of severe anterior knee pain and are significant contributors to the necessity for revision surgeries [7-9]. Patellar periprosthetic fractures (PPFs), after femoral periprosthetic fractures, constitute the second most frequent type of TKA periprosthetic fracture. These fractures occur in both unresurfaced and resurfaced patellae [10]. Less than half of PPF cases are attributable to direct trauma. Factors such as errors in surgical technique, inappropriate selection of implants, and a range of patient-specific risk factors play a critical role in their occurrence [11] (Figure 1).

Despite that PPFs are well described in numerous studies, the optimum management of the patella during TKA and the effective management of PPFs remains undefined [12,13]. Our study aims to underscore the severity of PPFs and to determine the significant role of the predisposing factors leading to this complication. In this study, a thorough review of the current literature was performed in PubMed, EMBASE, and Google Scholar databases investigating the epidemiology, predisposing factors, clinical presentation, classification, and diagnostic and treatment approaches of PPFs. This review intends to point out the most important factors that increase the risk of fracture and to summarize the management approach of patients with PPF.

## Review

Patella biomechanics

The patellofemoral joint is considered one of the most biomechanically complex joints in the human body due to its particular bone anatomy and the numerous musculo-capsulo-ligamentous structures that force dynamically on the patella [14]. The trochlear surface of the distal femur directly correlates with the posterior surface of the patella forming the diarthrodial plane patellofemoral joint [15]. The posterior surface of the patella can be segregated, by a vertical ridge, into two non-symmetric moieties consisting of a smaller and more steeply angled medial halve, and a larger and more shallowed lateral halve [16]. The Insall-Salvati ratio provides significant information about the sagittal plane patellar position concerning the increased risk of subluxation in patients with patella alta [17].

Since the 1970s, biomechanical modeling investigations focusing on normal knee joints have yielded insights into the elevated loads exerted on the patella during knee motion. This is particularly evident in a range of activities that involve high-flexion [18,19]. The patellar surface area that articulates depends on the knee joint motion, with the patellar articular surface becoming more proximal as the knee progresses through flexion. Biomechanically, the patellofemoral joint could be simulated to a pulley, which gradually receives increasing forces through the knee joint flexion, peaking at about 100-120 degrees [15,16]. Patellar loads seem to be equivalent to three times of body weight during squatting or stair climbing and one time of body weight during walking [20]. The unique anatomy of the patellofemoral joint, along with the particularly high loads it bears, renders it a complex joint. Any biomechanical dysfunction could induce severe pain, joint instability, and debilitated function, even in activities of daily living [14].

Epidemiology

Patellofemoral complications following TKA account for an incidence of up to 20% of TKAs, rendering them a primary indication for revision surgeries [8,9,21]. Periprosthetic patellar fractures constitute a relatively rare complication with a prevalence varying between 0.2% and 21%, making them the second most frequent TKA periprosthetic fracture after the femoral supracondylar periprosthetic fractures [13,22,23]. Ortiguera and Berry reported a prevalence of 0.68% of PPFs in their 30-year retrospective analysis of the Mayo Clinic registry in over 12,000 TKAs [12]. Patella periprosthetic fractures have been reported after both resurfaced and non-resurfaced patella techniques, however, resurfacing seems to predispose to fracture occurrence [23,24]. Chalidis et al. observed a higher incidence of PPF in resurfaced patellae (99.1% of PPFs) compared to non-resurfaced cases (0.9% of PPFs) in their systematic review [13].

Despite that PPFs may occur during TKA, most of the PPFs seem to be asymptomatic and detected during the early follow-up period, mainly within 2 years after total knee replacement [13,23]. A mean time from TKA to fracture occurrence of 18 months has been reported by Chalidis et al. [13]. Only less than half of the patients report a recent traumatic activity [8]. Furthermore, revision surgeries have been accused of a sharp increase in the incidence of PPFs compared to PPFs following primary TKAs [12,25,26]. Concurrently, complications such as the loosening of the patellar component, which are not uncommon, add complexity to the management of these cases [12,27].

As far as the sex distribution is concerned, several authors have observed a male prevalence and thus reported male gender as an independent risk factor of PPF occurrence [11,12,23]. Ortiguera and Berry observed an incidence of 1.01% of PPFs in male patients compared to 0.4% in females [12]. Accordingly, Parvizi et al. reported a male-to-female ratio of 2:1 [23]. This contrasts with the reports of several authors who observed a female predominance in other periprosthetic fractures around the knee joint, mainly attributed to osteoporosis [12,22,23,25]. Conversely, a few authors have noted a higher incidence of these fractures in male patients, suggesting different causative factors [28,29]. 

Risk factors

Multiple etiological factors have been implicated in increasing the risk for patellar fractures after total knee arthroplasty, including biomechanical, traumatic, vascular, and technical risk factors [12,30]. Patella periprosthetic fractures can result from both traumatic or non-traumatic factors. Although there is a trend of a non-traumatic background of PPFs, there is no established consensus in the literature that knee trauma is definitely associated with fracture occurrence [11-13,21,23,24,29,31]. Govil et al. described that 66% of the reported PPFs were associated with a traumatic event [29]. On the contrary, Chalidis et al. pointed out a markedly lower incidence (11.7%) of traumatic PPFs [13]. Both direct knee trauma and indirect lesions can result in PPF [12,24,32]. Ortiguera and Berry highlighted various indirect mechanisms of PPFs including overload of the knee joint, excessive flexion, deep seated positions, and eccentric muscle contraction [12]. Besides such cases, it is strongly recommended in the literature that PPF occurrence is multifactorial given the increased frequency of non-traumatic fractures associated with excessive resection, component loosening, surgical technique failures, and fatigue fractures due to bone loss [11,13,23,24]. Several studies have categorized such predisposing factors into patient-specific, surgical, and implant-related categories [11,13,23,24]. This is essential for understanding the fracture pattern and pathogenesis, which in turn is crucial to ensure the optimal management and treatment.

Patients’ data

High activity levels, particularly involving excessive or rapid knee flexion, along with obesity, seem to have a significant impact on the incidence of PPF [13,23,24]. Male gender is generally considered as a predisposing factor however, divergent results have been reported as far as gender predominance is concerned. [11,12,23,28,29]. Men demonstrate an increased incidence of these fractures, likely due to their generally higher engagement in vigorous physical activities or sports, which elevates the baseline activity level and consequently the risk of fracture [12,33]. Elderly patients preserve a greater risk of PPFs due to osteoporosis and a greater risk of falls [22]. Age has been underlined by Govil et al. as a predisposing factor for PPF [29]. On the contrary, Parvizi et al. mentioned that young patients’ greater activity levels can result in traumatic lesions and PPFs [23]. Moreover, comorbidities including rheumatoid arthritis, osteopenia, osteonecrosis, and extensive bone loss, seem to adversely affect the likelihood of fracture occurrence due to poor quality of the bone [11,13,23,24].

Surgical technique failure

Patella Devascularization and Osteonecrosis 

Patella blood supply is primarily provided via intraosseous and extraosseous vascular systems. These systems consist of six genicular arteries (extraosseous system) and the mid-patellar and polar vessels (intraosseous system) [34]. This peripatellar vascular system can suffer significant damage during TKA and the most commonly performed median parapatellar arthrotomy (MPA) [32]. Devascularization of the patella has been highlighted as a predisposing factor to patella osteonecrosis and increased risk of fracture in several studies [11,13,23,24].

During MPA, the medial genicular arteries are at risk of being compromised, necessitating reliance on the lateral genicular system to maintain the blood supply to the extensor apparatus. Lazaro et al. pointed out the existence of the oblique superior genicular artery (SGA), recommending a subvastus approach since MPA completely compromises SGA and medial genicular artery blood supply [35]. Additionally, preserving a peripatellar border of more than a centimeter during arthrotomy may preserve the peripatellar blood supply [35]. Besides that, patella hypovascularity is exacerbated by excessive fat pad excision, which can potentially damage the anastomosis between the inferior medial and lateral vessels. Consequently, it is recommended to limit the excision of the fat pad, as adequate exposure of the tibia can be achieved without necessitating extensive removal [36].

The blood supply of the patella is also sacrificed and associated when extensive lateral release is performed [11,13,35]. During MPA, the lateral release is a major concern, particularly in revision surgeries and valgus knees. Chalidis et al. observed that 51% of the PPF in their systematic review were associated with lateral release [13]. Extensive lateral release compromises lateral genicular artery (LGA) integrity. The LGA runs just peripheral to the lateral meniscus and hence, the typical complete lateral meniscectomy which is performed during TKA, can potentially divide LGA [32]. Lazaro et al. posited that the preservation of the lateral margin of the lateral meniscus significantly reduces the risk to the inferolateral genicular artery [35]. They advocate for maintaining a boundary around the patella exceeding 10-15 mm and propose that a lateral release, when conducted proximal to the joint line and distal to the superolateral edge of the patella, is associated with enhanced safety [35].

Patella vascularization can also be compromised during eversion or retraction of the patella during TKA. Hempfing et al. pointed out a markable reduction of the peripatellar blood supply associated with patella eversion and knee flexion of 100° [37]. However, the authors did not mention any association between soft tissue release and devascularization [37]. Accordingly, Stoffel et al. observed less than 50% decrease in blood supply when slight retraction was performed [38]. This decrease was estimated to be almost 90% during eversion, as measured with the Doppler ultrasound [38]. Regardless of the choice of the surgical approach or the mandatory soft tissue release, vascular integrity can be compromised when bone cement is used. Several authors considered that factors including byproducts of polymethylmethacrylate cement polymerization and the heat generated during this process have been implicated in causing thermal necrosis and additional vascular damage [11,13,24,29].

Patella Resection/Resurfacing

The average thickness of the male patella is 25 mm, whereas that of the female is 22 mm [39]. During TKA, patella thickness should be restored to native. Overstuffing of the joint with a thicker patella has been accused of lateral subluxation and flexion gap. On the contrary, overresection increases anterior stress forces during flexion and predisposes to stress fracture [13]. The majority of authors consider in favor of a minimal intraoperative patella resection, removing the least possible articular segment and retaining the peripheral cortex of both the medial and lateral facets and adequate anterior cortex [13,30,31,40,41].

Generally, resection of the patella is considered to increase the risk of fracture. Chalidis et al. reported a markedly higher rate of PPF in resurfaced patellae (577 cases, 99.1%) compared to PPF in non-resurfaced techniques (5 cases, 0.9%) [13]. Conversely, Ritter et al. did not observe any significant association between patella thickness and PPF [27]. Windsor et al. and Bourne pointed out that over-resection constitutes a significant risk factor for PPF [31,42]. The authors suggest that patella thickness should be at least 10-15 mm [31,42]. Reuben et al. reported increased anterior strain forces when patella thickness is less than 15 mm [43]. Notably, Wilson et al. observed great long-term survivorship rates and suggested resurfacing of thin native patellae [44].

Currently, there is no consensus in the literature regarding the efficiency of patella resection. Although resection may lead to a limited number of complications, it is a cost-effective procedure that effectively reduces the rates of re-intervention. Furthermore, current evidence does not indicate a clinically significant difference in the functional result when comparing resurfacing techniques [44,45]. Existing research underscores the significance of achieving optimal symmetry in patellar resurfacing. Additionally, similar outcomes are attainable across various patellar cutting techniques in the literature [46,47].

Implant-associated risk factors

Implant Alignment

Implant positioning and alignment are crucial in ensuring optimal patellar tracking within the patellofemoral joint [48]. Misalignment of femoral and tibial implants, as well as the patellar prosthesis, can lead to significant increased and eccentric forces among the extensor apparatus and patella maltracking, increasing the risk of fracture [49]. While traditional surgical approaches have favored medialization of the patellar prosthesis to decrease lateral retinacular strain and reduce the need for lateral release, the choice and placement of the implant should be highly individualized, taking into account the unique characteristics of each patient [50,51].

Rotational or angular tibia of femoral component malalignment sharply increases traction forces among the extensor apparatus predisposing to PPF. Excessive internal rotation of either the femoral or tibial component has been associated with increased retropatella stress and significant patellofemoral complications [52,53]. Increasing the anteroposterior diameter of the joint as well as a flexed femoral implant also constitutes a risk factor for PPF [11,23,24,29]. Seo et al. yielded an association of PPF with decreased length of the patella tendon and therefore the Insall-Salvati ratio and severe preoperative deformity [54]. Figgie et al. reported that minor implant misalignment was associated with PPF without loosening of the patella component and almost all were managed conservatively [55]. Conversely, severe malpositioning resulted in complex PPF with loosening of the implant necessitating surgical treatment in several cases [55]. 

Patella Implant Design and Material

The choice of material for the patellar prosthesis is critical in TKA outcomes. Traditional polyethylene gamma sterilized implants have been implicated­ in the long-term oxidative degradation of patellar components [56,57]. The most current high-cross-linked polyethylene have not demonstrated definite benefits, since they present degraded mechanical strength and fatigue [58,59]. However, some authors report that high-cross-linked polyethylene shows increased resistance to damage, attributed to its lower friction levels [60,61].

Patella periprosthetic fracture seems also to be significantly influenced by specific patellar component designs. Metal-backed components accounted for limited polyethylene damage. However, such components resulted in severe strain among the cement-component and/or bone-component interface encompassing a higher risk of loosening and PPF [32]. Healy et al. observed significantly greater rates of PPF in metal-backed patella implants compared to cemented all-polyethylene patellae [9]. Bayley et al. disclosed that metal-backed patellar implants result in more stiffness in the joint and therefore increased loads and stress on the augmented interface, increasing the risk of PPF [62]. Moreover, implants with a large central peg, increase anterior strain predisposing to a higher risk of PPF [13,23,24,27]. Generally, mainly metal-backed uncemented implants and those with large central pegs are considered to result in an increased risk of PPF. Hence, modern components usually consist of cemented all-polyethylene components with 3 smaller pegs, aiming to achieve lower levels of anterior strain and potentially reduce the risk of fractures [11,13,23,24]. Larson et al. observed an almost double incidence of PPF (4.7% compared to 2.1%) in single-peg components compared to 3-peg implants [63].

Historically, cemented patellar components have been the preferred choice in TKA, owing to their impressive survival rates and excellent clinical outcomes [64,65]. However, in recent years, there has been a growing trend towards the use of modern cementless implants. These cementless options have demonstrated equivalent results to cemented implants, with minimal complication rates, suggesting that the cementless technique can be a safe and effective alternative [66,67]. Despite these promising findings, some concerns remain. Chan and Giori reported a significant rate of more than 20% of patella fractures within 5 years post-TKA, highlighting the need for further research in this area [68].

Diagnostic and treatment approach and reported outcomes

Patients with PPF are often asymptomatic and PPF is detected during regular follow-up. Chalidis et al. mentioned that more than 85% of the studied patients were asymptomatic and fractures were diagnosed in a mean time of 18 months after TKA [13]. However, several studies have reported manifestations such as patellar tenderness and swelling, anterior knee pain, knee instability, and lag of the extensor apparatus, all of which signify the imminent detection of fractures [24,29]. Currently, diagnosis of PPF is mainly based on anteroposterior, lateral, and Merchant views X-rays [22,24,29]. A computed tomography (CT) scan as well as a bone scan with Technetium 99 (Tc-99m) have been also introduced in the diagnostic process establishing crucial specifics concerning the fracture pattern, implant loosening, and discriminate occult or old fractures [24,29]. However, it is crucial to evaluate previous X-rays and assess if the loosening of the implant was already presented before the fracture detection, even if the time discrimination of the fracture or the component loosening is difficult and not usually feasible [27].

Several classification systems have been proposed for PPF, mainly evaluating the extensor apparatus integrity, the stability or loosening of the patellar component, the quality of the remaining patellar bone stock, and the fracture pattern. Although there is no established and globally adopted classification system, to date, Ortiguera’s and Berry’s classification and this proposed by Golberg et al. are the most widely accepted and used for the discrimination of PPF [12,28]. Notably, Windsor et al. classified non-traumatic fatigue PPF considering the fracture pattern and associated such PPF with the responsible indirect mechanism [31]. The authors divided PPF into vertical, transverse, and comminuted-displaced and mentioned that horizontal fractures are associated with patella maltracking and dislocation. Vertical fractures, often occurring at the patella fixation holes, are distinct from comminuted and displaced fractures, which typically result from a combination of transverse and vertical fractures [31].

Treatment considerations of PPF are mainly attributed to the integrity of the extensor apparatus, the quality of the remaining bone stock, the stability of the implant, and the fracture displacement and comminution [13]. Therefore, treatment options consist of conservative management with a brace or cast immobilized in extension for 4-6 weeks with partial or full weight bearing allowed [11-13,21,23,28,31]. Surgical treatment includes open reduction and internal fixation (ORIF), revision of the patellar component, partial or total patellectomy with repair of the patellar or quadriceps tendon, and extensor apparatus reconstruction [11-13,21,23,24,28,29,69-71].

Despite that several classification systems and treatment algorithms have been proposed, there is a general trend and most authors advocate in favor of a conservative treatment of PPF when the extensor mechanism is intact and the patella component is stable [13,23,29]. Chalidis et al. reported that 68% of the studied patients in their systematic review were treated conservatively and 31% were treated by operative means [13]. Conservative treatment is preferred when indicated since operative approach and revision surgeries carry a significant risk of infection and complications. Chalidis et al. pointed out an average infection rate of 19.2% after surgical treatment of PPF [13]. Generally, conservative treatment of PPF with an intact extensor mechanism and stable implant results in good functional outcomes without instability of the patella and pain however, a minor lag in extension of about 5° is mentioned in few studies [12,13,21,23,24,29,72]. Keating et al. observed excellent outcomes in 21 type I, according to Ortiguera and Berry classification, PPF who underwent conservative treatment. The authors noted negligible pain, an average flexion of 120°, and a mean lag in extension less than 5° [72]. Although fibrous union or union is observed in several cases, pain and discomforts are rare, knee function is restored and fair outcomes are reported [11-13]. 

Patella periprosthetic fractures with disruption of the extensor apparatus mostly require surgical treatment regardless of the stability of the patellar implants as knee function impairment is highly compromised [13]. Although several surgical options have been described including ORIF, partial or total patellectomy, and extensor apparatus repair or reconstruction, the reported outcomes are poor and the mentioned complication rate is considerably high [5,12,13,23,30,40,72]. Chalidis et al. observed poor outcomes due to failed ORIF in more than 90% of the studied patients [13]. Failure of ORIF, infection, and non-union of the fracture have been reported at significant rates mainly attributed to the inadequate blood supply and therefore the potential impotence of healing of the fracture fragments [72,73]. Hence ORIF is not often a simple procedure, whereas partial patellectomy with extensor apparatus repair is recommended by several authors when poor-quality small fragments are detected and afterwards removed. However, patellectomy may lead to weakness of the quadriceps and subsequent lag in extension [34,41,74]. 

Loosening of the patellar implant constitutes an indication of surgical treatment and removal of the loose component [11-13,24]. The optimal treatment after the removal is highly individualized depending on the residual bone stock thickness and bone quality [12,13,71]. In cases of significant bone defect, resection arthroplasty with total or partial patellectomy constitutes the most recommended surgical option [12,13,71,73]. However, where residual bone thickness of more than 10 mm remained, revision of the component and patelloplasty is the most reasonable surgical approach [12,13,75]. Regardless of the selected treatment approach, the complication rate after surgical treatment of type III PPF, according to Ortiguera and Berry classification, is high. Ortiguera et al. observed a 29% complication rate in surgically treated patients with type III PPF [12]. The authors also reported a more than 10% reoperation rate and more than half of the patients were symptomatic during the last follow-up [12]. Moreover, Parvizi et al. mentioned that reoperation was addressed in 2 out of 3 patients with type III PPF [23]. Lastly, Windsor et al. provided their treatment approach according to the fracture pattern and displacement [31]. Conservative treatment was suggested for comminuted, transverse, and vertical fractures where displacement was less than 2 cm. Conversely, surgical treatment was indicated when quadriceps weakness and loss in extension were detected as well as in transverse fractures with severe comminution and displacement of more than 2 cm [31].

## Conclusions

Patellar periprosthetic fractures (PPF), while infrequent, present significant therapeutic challenges, with a high complication rate, contributing to poor outcomes after total knee arthroplasty (TKA). Patients’ education is crucial in reducing their exposure to predisposing factors and diminishing both the incidence and complexity of managing these fractures. Although risk factors related to patients’ specifics are well established, the significant rate of complications following surgical treatment of PPF determines the necessity of ongoing research and refinement of the surgical approaches. Patellar manipulation during TKA should be refined taking into numerous factors. The surgical management of PPFs should aim for pain-free and functional knee joints, ensuring both the restoration of the extensor apparatus and the stability of the prosthesis. Currently, conservative management is predominantly recommended, reflecting the significant incidence and severity of postoperative complications.
